# Assessment of MultiLocus Sequence Analysis As a Valuable Tool for the Classification of the Genus *Salinivibrio*

**DOI:** 10.3389/fmicb.2017.01107

**Published:** 2017-06-22

**Authors:** Clara López-Hermoso, Rafael R. de la Haba, Cristina Sánchez-Porro, R. Thane Papke, Antonio Ventosa

**Affiliations:** ^1^Department of Microbiology and Parasitology, Faculty of Pharmacy, University of Sevilla Sevilla, Spain; ^2^Department of Molecular and Cell Biology, University of Connecticut, Storrs CT, United States

**Keywords:** *Salinivibrio*, halophilic bacteria, MLSA, DNA–DNA hybridization, genomic fingerprinting

## Abstract

The genus *Salinivibrio* includes obligatory halophilic bacteria and is commonly isolated from hypersaline habitats and salted food products. They grow optimally between 7.5 and 10% salts and are facultative anaerobes. Currently, this genus comprises four species, one of them, *S. costicola*, with three subspecies. In this study we isolated and characterized an additional 70 strains from solar salterns located in different locations. Comparative 16S rRNA gene sequence analysis identified these strains as belonging to the genus *Salinivibrio* but could not differentiate strains into species-like groups. To achieve finer phylogenetic resolution, we carried out a MultiLocus Sequence Analysis (MLSA) of the new isolates and the type strains of the species of *Salinivibrio* based on the individual as well as concatenated sequences of four housekeeping genes: *gyrB*, *recA*, *rpoA*, and *rpoD*. The strains formed four clearly differentiated species-like clusters called phylogroups. All of the known type and subspecies strains were associated with one of these clusters except *S. sharmensis*. One phylogroup had no previously described species coupled to it. Further DNA–DNA hybridization (DDH) experiments with selected representative strains from these phylogroups permitted us to validate the MLSA study, correlating the species level defined by the DDH (70%) with a 97% cut-off for the concatenated MLSA gene sequences. Based on these criteria, the novel strains forming phylogroup 1 could constitute a new species while strains constructing the other three phylogroups are members of previously recognized *Salinivibrio* species. *S. costicola* subsp. *vallismortis* co-occurs with *S. proteolyticus* in phylogroup 4, and separately from other *S. costicola* strains, indicating its need for reclassification. On the other hand, genome fingerprinting analysis showed that the environmental strains do not form clonal populations and did not cluster according to their site of cultivation. In future studies regarding the classification and identification of new *Salinivibrio* strains we recommend the following strategy: (i) initial partial sequencing of the 16S rRNA gene for genus-level identification; (ii) sequencing and concatenation of the four before mentioned housekeeping genes for species-level discrimination; (iii) DDH experiments, only required when the concatenated MLSA similarity values among a new isolate and other *Salinivibrio* strains are above the 97% cut-off.

## Introduction

The genus *Salinivibrio* constitutes a phylogenetic lineage within the *Vibrionaceae* according to 16S rRNA gene sequence analysis. This genus was proposed by Mellado et al. (1996), who reclassified the species *Vibrio costicola* due to its substantial differences in phylogenetic, phenotypic and genotypic characteristics with respect to other *Vibrio* species. Currently, *Salinivibrio* consists of four species, one of them with three subspecies, *S. costicola* subsp. *costicola* (Mellado et al., 1996), *S.* *costicola* subsp. *vallismortis* (Huang et al., 2000), *S. costicola* subsp. *alcaliphilus* (Romano et al., 2005), *Salinivibrio proteolyticus* (Amoozegar et al., 2008), *S.* *siamensis* (Chamroensaksri et al., 2009), and *S. sharmensis* (Romano et al., 2011). The type species of the genus is *S. costicola* subsp. *costicola.* The species of this genus have been isolated from salted meats, brines and other hypersaline environments. A recent study based on comparative genomics of three strains belonging to the genus *Salinivibrio* has been reported, showing that they may constitute a new taxon, but they have not been formally proposed as a new species of this genus. A xanthorhodopsin gene cluster, which could be linked to a light-based energy production system, was observed in these genomes (Gorriti et al., 2014).

The phylogeny of the family *Vibrionaceae* based on a 16S rRNA gene approach is confusing since it is not always possible to differentiate among closely related members of this family mainly because of the high levels of conservation of this phylogenetic marker. Several studies have shown that the 16S rRNA gene lacks resolution for discrimination between nearly related bacterial species (Santos and Ochman, 2004; Staley, 2006; Gao et al., 2016). The classification of the genus *Salinivibrio* needs reappraisal since the three subspecies of *S. costicola* do not form a monophyletic group; this conclusion is supported by previous studies carried out in the genus *Salinivibrio* that have shown one of the subspecies, *S. costicola* subsp. *vallismortis*, is not related to the other two, but forms a monophyletic group with another species of the genus, *S. proteolyticus* (Amoozegar et al., 2008; Chamroensaksri et al., 2009; Romano et al., 2011; Gorriti et al., 2014).

Multilocus sequence analysis (MLSA), i.e., concatenating the sequences of several protein-encoding gene fragments, provides a more robust tree topology and an improved understanding of speciation events in comparison to a tree based only on 16S rRNA gene sequences (Thompson et al., 2005, 2007; Pascual et al., 2010; Sawabe et al., 2013). In order to carry out an MLSA study, several housekeeping loci are sequenced and compared, and evolutionary relationships among the taxa are established. One important advantage of this methodology is the database availability of gene or genomic sequences, in contrast to DNA–DNA reassociation data, the taxonomic standard for circumscribing new species. However, the usefulness of MLSA for describing and circumscribing bacterial species needs to be validated on a case-by-case basis, demonstrating that there is a sufficient degree of congruence between MLSA and DNA–DNA reassociation data (Pascual et al., 2010). *Salinivibrio* is an ideal candidate for MLSA with a low number of species to be analyzed which allows in-depth study of the inter- and intraspecies phylogenetic relationships.

PCR-based genomic fingerprinting methods can provide more refined diversity resolution than 16S rRNA gene or MLSA sequencing for differentiation at the strain level. Genomic fingerprints generated by PCR with primers binding to interspersed repetitive sequences (rep-PCR) (Versalovic et al., 1994) give the highest level of taxonomic resolution currently achievable by PCR methods (de Brujin, 1992; Laguerre et al., 1996; Abdollahzadeh and Zolfaghari, 2014). Besides, a high degree of reproducibility of the rep-PCR technique has also been demonstrated (Clark et al., 1998; Momeni et al., 2015; Jarocki et al., 2016). However, this method’s ability to detect genomic variation or circumscribe *Salinivibrio* species has not been evaluated.

The aim of the present study was to refine the understanding of the phylogenetic relationships of the species and subspecies in the genus *Salinivibrio* and to help to clarify the current classification of this genus. For that purpose we used an MLSA approach based on *gyrB*, *recA*, *rpoA*, and *rpoD* gene sequences as an alternative to the 16S rRNA gene-based phylogeny. A total of 6 type strains and 70 representative new isolates of *Salinivibrio* were used here. This MLSA scheme was validated by comparison with DNA–DNA hybridization (DDH) studies in order to replace the latter for species delineation in the genus *Salinivibrio*. In addition, we used a PCR-based genome fingerprinting to compare genetic variation and differentiate clonal strains.

## Materials and Methods

### Sampling Sites

Samples were collected from different solar salterns, from Spain: Isla Bacuta (37°14′50.60′′N, 6°58′0.96′′O); Aragonesas (37°15′34.89′′N, 6°58′31.98′′O); Isla Cristina (37°12′51.36′′N, 7°19′20.34′′O); La Malahá (37° 6′12.93′′N, 3°43′16.07′′O); Es Trenc (39°21′6.80′′N, 3°0′24.99′′E); Bañaderos (27°50′14.68′′N, 15°25′18.61′′O); Bras del Port (38°11′48.44′′N, 0°35′13.22′′O), and from Puerto Rico: Cabo Rojo (17°57′9.74′′N, 67°11′42.38′′O). The salinity and pH of the samples are shown in the Supplementary Table 1.

### Strains and Culture Conditions

In order to isolate a collection of strains of the genus *Salinivibrio*, 0.1 ml of each water sample was inoculated on SW plates. The SW medium contained (l^-1^): yeast extract (Bacto^TM^) 5 g, MgCl_2_⋅6H_2_O 9.75 g, NaCl 58.5 g, MgSO_4_⋅7H_2_O 15.25 g, KCl 1.5 g, CaCl_2_ 0.25 g, NaHCO_3_ 0.05 g and NaBr 0.175 g. The pH was adjusted to 7.2–7.4 with 1 M KOH. Due to the fact that they are facultatively anaerobic bacteria, the plates were incubated at 37°C in an anaerobic chamber (Oxoid) for 1–2 weeks. Once the incubation period was complete, the microorganisms were observed under a microscope to select those that had a curved or S-shaped appearance. These were subcultured in pure culture in the same isolation medium, but grown in the presence of oxygen. A total of 170 strains were screened using amplification and partial sequencing of the 16S rRNA gene in order to determine the phylogenetic position of each of them and to confirm that they were strains belonging to the genus *Salinivibrio*. Finally, a collection of 70 new strains belonging to this genus was selected.

A total of 76 strains were used in this study (Supplementary Table 2), including type and environmental strains. These strains were routinely cultivated under aerobic conditions in SW medium at 37°C for 24–48 h and were preserved either on solid slant tubes at room temperature and also as 20% glycerol suspensions at -80°C.

### DNA Preparation

Genomic DNA from each culture was obtained by the method of Marmur (1961) and, after quantification, its quality was evaluated using a Nanodrop spectrophotometer ND-1000 at 260/280 nm. Finally, the genomic DNA was diluted with 1 M Tris/HCl to a final concentration of 20 ng μl^-1^ for subsequent PCR analysis.

### PCR Amplification and Sequencing of 16S rRNA and Housekeeping Genes

Initially, the 16S rRNA gene was amplified by PCR and sequenced using the forward primer 16F27 to obtain the partial sequence of the environmental strains. Once confirmed that they belonged to the genus *Salinivibrio*, with sequences showing a percentage of similarities higher than 94%, the reverse primer 16R1488 was also used in order to obtain the complete 16S rRNA gene sequence. Additionally, the intermediate primers 16R343, 16F530 and 16R530 were also employed to obtain a high confidence gene sequence (Mellado et al., 1995; **Table 1**).

**Table 1:** Oligonucleotide primers used for PCR amplification and sequencing.

Gene	Primer	Sequence (5′→ 3′)	Position^∗^	Reference
16S rRNA	16F27	AGA GTT TGA TCM TGG CTC AG	8–27	Mellado et al. (1995)
	16F530	GTG CCA GCA GCC GCG G	515–530	Mellado et al. (1995)
	16R343	ACT GCT GCC TCC CGT A	358–343	Mellado et al. (1995)
	16R530	CCG CGG CTG CTG GCA C	545–530	This study
	16R1488	CGG TTA CT TGT TAG GAC TTC ACC	1511–1488	Mellado et al. (1995)
*gyrB*	gyrB1626F	TGT AAA ACG ACG GCC AGT CAA GAG CAG TAC ATY AAA GAY G	1626–1664	This study
	gyrB2230R	CAG GAA ACA GCT ATG AC TC TGG GTT CATCTC RCC	2246–2239	This study
*recA*	recA-01-F	TGA RAA RCA RTT YGG TAA AGG	54–74	Thompson et al. (2008)
	recA-02-R	TCR CNT TRT AGC TRT ACC	889–872	Thompson et al. (2008)
*rpoA*	rpoA-01-F	ATG CAG GGT TCT GTD ACA G	1–19	Thompson et al. (2005)
	rpoA-03-R	GHG GCC ART TTT CHA RRC GC	967–947	Thompson et al. (2005)
*rpoD*	rpoD-70-F	ACG ACT GAC CCG GTA CGC ATG TAY	280–303	Pascual et al. (2010)
	rpoD-70-R	ATA GAA ATA ACC AGA CGT AAG TTN GCY TCN ACC ATY TCY TTY T	1169–1127	Pascual et al. (2010)

*^∗^Binding-position numbering is based on the full-length gene sequences in the Escherichia coli K-12 genome (Blattner et al., 1997; Riley et al., 2006).*

The following genes were partially amplified and sequenced: *gyrB* (DNA gyrase, B subunit); *recA* (recombinase A); *rpoA* (RNA polymerase, α subunit); and *rpoD* (RNA polymerase, β subunit). The primers that were developed and used in this study are listed in **Table 1**. These housekeeping genes were selected based on previous studies of orthologous genes successfully used for MLSA in the phylogenetically related genus *Vibrio*. Primers for *recA* were developed by Thompson et al. (2008) and Pascual et al. (2010); primers for *rpoA* by Thompson et al. (2005) and primers for *rpoD* by Pascual et al. (2010). Primers for *gyrB* were designed in this work (additional information available in Supplementary Material).

PCR amplification was carried out in a 50 μl reaction mixture with the following composition: 2.5 μl forward primer (12 μM), 2.5 μl reverse primer (12 μM), 8.0 μl dNTPs (1.25 μl each), 2.5 μl MgCl_2_ (25 mM), 5.0 μl PCR buffer (10X), 0.5 μl *Taq* polymerase (5 U μl^-1^; iNtRON Biotechnology) and 5.0 μl template DNA (50 ng μl^-1^). A Mastercycler Ep Thermocycler (Eppendorf) was employed for amplification with cycling conditions set to: for 16S rRNA gene [5 min at 95°C; 25 × (1 min at 94°C, 1 min at 50°C, 2 min at 72°C); 10 min at 72°C]; for *gyrB* [5 min at 95°C; 35 × (1 min at 94°C, 1 min at 59°C, 1 min 30 s at 68°C); 10 min at 72°C]; and for *recA*, *rpoA*, and *rpoD* [5 min at 95°C; 3 × (1 min at 95°C; 2 min 15 s at 55°C; 1 min 15 s at 72°C); 30 × (30 s at 95°C; 1 min 15 s at 55°C; 1 min 15 s at 72°C); 7 min at 72°C]. The PCR bands were visualized after electrophoresis in agarose gel (1% w/v) prepared with ethidium bromide (0.625 μg ml^-1^); in order to calculate the molecular weight of the amplicons, a molecular mass marker (iNtRON Biotechnology) was used. Then, the amplicons were purificated by means of the FavorPrep GEL/PCR Purification Mini Kit (Favorgen Biotech) and subsequently sequenced by the dideoxynucleotide chain-termination method using the same primers as those for the amplification but they were diluted 1- and 2-fold (1 μM).

### Phylogenetic Data Analysis

The sequences obtained from 16S rRNA gene and housekeeping genes were assembled by using ChromasPro software (Technelysium Pty) and edited to resolve ambiguous positions. Each gene sequence established in this study was subjected to nucleotide-nucleotide BLAST analysis to support the identity of the gene. Multiple sequence alignments were made using CLUSTAL_X 2.1 (Larkin et al., 2007) and corrected by visual inspection using BioEdit (Hall, 1999) taking into account the corresponding amino acid alignments for protein-encoding genes. The total length of the alignments used were: 623 bp for *gyrB* gene, 771 bp for *recA* gene, 825 bp for *rpoA* gene and 741 bp for *rpoD* gene. Phylogenetic analysis was performed using MEGA 5 (Tamura et al., 2011) for maximum-parsimony (MP) and neighbour-joining (NJ) methods and PhyML (Guindon and Gascuel, 2003) for the maximum-likelihood (ML) (Felsenstein, 1981) method. NJ analyses were carried out using Jukes-Cantor substitution model (Felsenstein, 1991). MP analyses were performed using a heuristic search option. In the case of the ML method, the General Time Reversible model was selected and the rate matrix, the base frequencies, the invariable site proportion and the gamma distribution were determined via likelihood. For NJ, MP and ML phylogenetic tree branch support estimation, 1000 pseudoreplicates were calculated to obtain the corresponding bootstrap values (Felsenstein, 1985).

### Descriptive Analyses

The number of polymorphic sites and mutations, nucleotide diversity per site (ð), average pairwise nucleotide differences per sequence (k) and Tajima’s *D* test were separately calculated using DnaSP version 5.1 (Librado and Rozas, 2009).

### DNA–DNA Hybridization and Correlation Studies

DNA–DNA hybridization studies were carried out following a competition procedure in a nitrocelulose membrane (Johnson, 1994) as described elsewhere (Arahal et al., 2001a,b; León et al., 2016). The range of hybridization temperature used was between 51.8 and 55.0°C, which is within the limit of validity for the filter method used in this study (De Ley and Tijtgat, 1970). The percentage of DDH was calculated following the methodology described by Johnson (1994). All experiments were carried out in triplicate and the results shown are the mean values. The interpretation is according to Wayne et al. (1987) where it has been established that strains belonging to the same species should show values of DDH at or above 70%. DDH was also calculated *in silico* by the Genome-to-Genome Distance Calculator (GGDC 2.0) using the BLAST+ method (Meier-Kolthoff et al., 2013).

### Genomic Fingerprinting

Repetitive Extragenic Palindromic sequence-based Polymerase Chain Reaction (Rep-PCR) genomic fingerprinting was carried out as previously described (Vinuesa et al., 1998; Rademaker et al., 2000). In this study, the primer BOX-A1R was used to amplify the banding patterns. Amplification conditions were equal for all the strains tested to enable comparison between the banding patterns and the experiments were performed in triplicate to guarantee the obtaining of a repeatable banding profile. Each DNA sample was diluted to 25 ng μl^-1^ and amplified using the following reaction mixture: 4.5 μl Phire reaction buffer (5X), 1.90 μl DMSO, 1 μl dNTPs mix (10 mM), 1 μl primer BOX-A1R (5′-CTACGGCAAGGCGACGCTGACG-3′), 0.4 μl Phire Hot Start II DNA polymerase, 1 μl template DNA, and 15.2 μl H_2_O. The thermocycler program used was: 30 cycles (95°C for 2 min, 94°C for 3 s, 92°C for 3s, 40°C for 1 min) and a final extension of 65°C for 8 min. Rademaker et al.’s (2000) protocol was modified with the aim to increasing the resolving power of the method producing as many non-specific bands as possible for each sample.

After amplification PCR tubes were maintained at 4°C before electrophoresis. 1.5% (w/v) agarose gels were used and run at 12 V for 16 h at 4°C with the goal of producing crisp bands easily distinguishable by the analysis software. The gels were stained with ethidium bromide prior to imaging (Ram Mohan et al., 2014). For each gel a digital image was obtained using a GelDoc (UVP) system. These images were analyzed using the program Phoretix 1D Pro. Banding patterns were standardized for cross gel comparisons by calibrating Rf lines on the individual gels. Similarities between the banding patterns were analyzed using the Pearson correlation coefficient. The final dendrogram was created using Phoretix 1D Pro with UPGMA clustering algorithm (Ram Mohan et al., 2014).

## Results and Discussion

### 16S rRNA Gene Sequence Analysis

**FIGURE 1:**
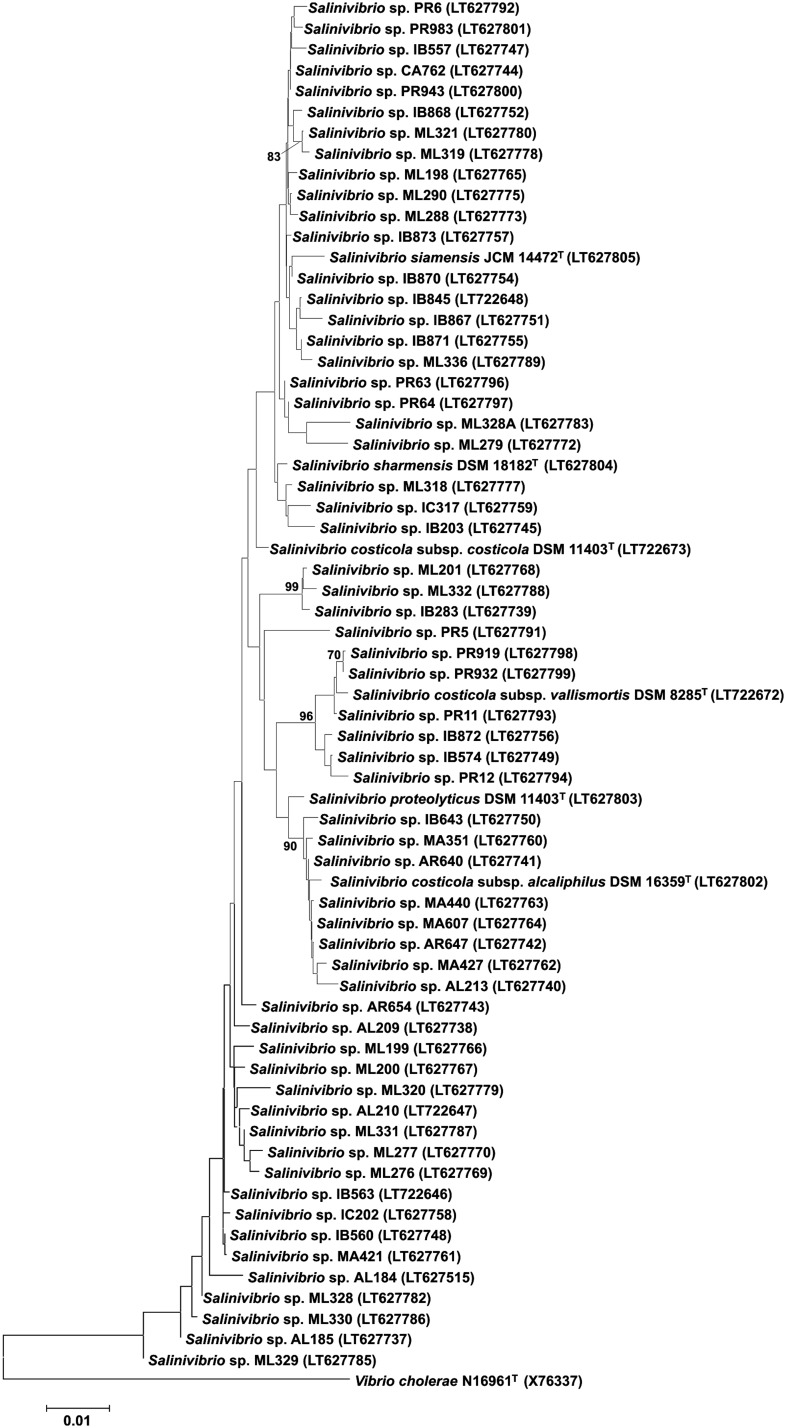
Neighbor-joining tree based on nearly complete 16S rRNA gene sequences showing the relationships between isolated and type strains of the genus *Salinivibrio*. Filled circles indicate nodes that were also recovered in maximum-parsimony and maximum-likelihood trees based on the same sequences. Numbers at nodes are bootstrap support values (percentages) based on analyses of 1000 resampled datasets; only values above 70% are shown. The GenBank/EMBL/DDBJ accession number of each sequence is shown in parentheses. Bar, 0.01 nt changes per position. *Vibrio cholerae* N16961^T^ was used as an outgroup.

After sequencing the partial 16S rRNA gene of the 170 strains initially isolated, we confirmed that 70 strains belonged to the genus *Salinivibrio*, and we selected these strains for further studies. Almost-complete 16S rRNA gene sequences (1209–1470 bp) of the 70 new strains selected for this study were used for the phylogenetic analysis. The type strains were also sequenced to check if our sequences were the same as the deposited ones. In all cases the sequences were the same and with the same length as those deposited, except in the case of *S. proteolyticus* where our sequence was longer than that deposited (1510 vs. 1489 bp). The analyses showed that 16S rRNA gene sequence similarities between strains ranged from 96.3 to 100%. Only three strains were between 96.3 and 97%. The range between type strains of this genus was from 97.6 to 100% and similarities between type strains and isolated strains ranged from 96.3 to 100%; the majority of the similarity values were higher than 97%. It was also shown that the bootstrap support for the 16S rRNA gene tree was generally below 70%, especially for the deeper nodes, and therefore it was not possible to distinguish reliable or robust phylogroups (**Figure 1**). Several previous studies have shown the limitations of the 16S rRNA gene sequence as a single phylogenetic marker for comparative phylogenetic studies (Thompson et al., 2005, 2008; Pascual et al., 2010) and our results corroborate their findings. Additionally, the cut-off for typical species delineation is 97% sequence similarity, and our results demonstrate that the entire 16S rRNA diversity within this genus is comparable to that value, i.e., above or equal to 96.3%, corroborating that the 16S rRNA gene does not contain sufficient variation to differentiate species within *Salinivibrio*. Since MLSA has been suggested as the best alternative approach to the 16S rRNA gene-based phylogeny (Pascual et al., 2010; Papke et al., 2011; Sawabe et al., 2013; Gao et al., 2016) we decided to apply that technique for *Salinivibrio* phylogenetic analysis and comparisons.

### MLSA Based on Concatenated Gene Sequences

This MLSA study was based on the sequence analysis of the housekeeping genes *gyrB*, *recA*, *rpoA*, and *rpoD*. The decision to include these genes was based on previous studies on the genus *Vibrio* (Thompson et al., 2005, 2008; Pascual et al., 2010). We tested additional loci, *pyrH*, *secA*, and *atpA*, but an amplification product was not obtained for the most of the strains, so these results were not included in this study. Almost all partial gene sequences of the genes *gyrB*, *recA*, *rpoA*, and *rpoD* were obtained for this study (Supplementary Table 2).

The concatenated gene sequence analysis enhances the phylogenetic reconstruction quality and also optimizes the taxonomic structure resolution. Besides, more informative data are analyzed and the weight of recombination events is minimized. In phylogenetic studies, the use of a minimum number of genes is crucial to optimize time and cost. This is of particular importance when establishing species and elucidating population structure and evolution in a super bacterial taxon such as in the case of the family *Vibrionaceae*, which has more than 140 species (Sawabe et al., 2013).

**FIGURE 2:**
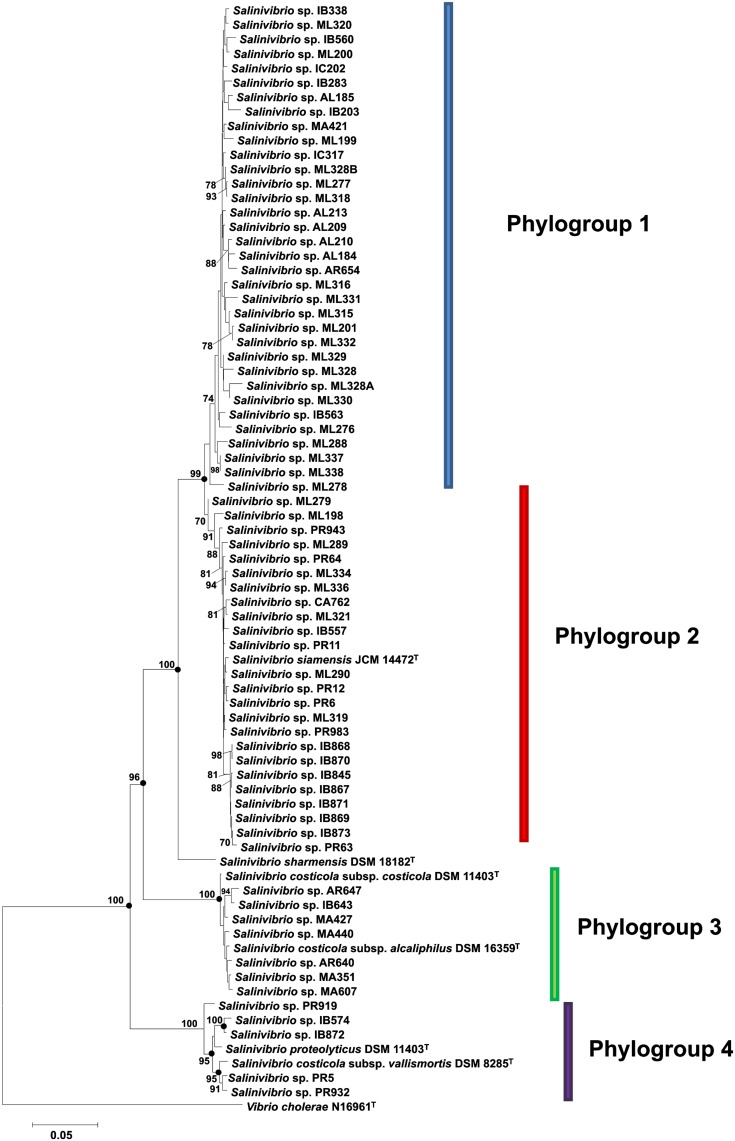
Phylogenetic reconstruction of *Salinivibrio* strains based on concatenated *gyrB*, *recA*, *rpoA*, and *rpoD* gene sequences. The tree is based on 2981 nt of common sequence. Analysis was done using the neighbor-joining method. Circles indicate branches that were supported by NJ, MP and ML algorithms. Only bootstrap values above 70% are shown (1000 replications) at branches points. Bar, 0.05 expected nucleotide substitutions per site. *V. cholerae* N16961^T^ was used as an outgroup.

Concatenation of the sequences of the four genes (*gyrB*, *recA*, *rpoA*, and *rpoD*) yielded an alignment of 2981 nt. The sequence similarities among the 76 tested strains ranged from 80.0 to 100%. The phylogenetic trees generated from the concatenated four-gene nucleotide sequences (**Figure 2**) showed well or very well defined phylogroups. The results of this MLSA study demonstrated that the concatenated MLSA phylogeny was the best at differentiating phylogroups.

The neighbor-joining *gyrB*-*recA*-*rpoA*-*rpoD* concatenated tree (**Figure 2**) showed that the 76 strains constituted four different phylogroups, with only one strain belonging to the species *S. sharmensis* (with a high bootstrap value of 100%), which cannot be included within any phylogroup since we could not isolate any strain belonging to this species and, therefore, *S. sharmensis* likely represents a unique phylotype. This tree showed that strains included in the phylogroups 3 and 4 are clearly separated from the rest, showing very high bootstrap support (100% for both phylogroups). In the case of phylogroups 1 and 2, they are supported by a high bootstrap value (99.0%) for the branch containing both phylogroups, so they might form a single phylogroup. However, it seems also reasonable that all these strains can be assigned to two different phylogroups since the bootstrap value supporting the phylogroup 2 is 70%, so it constitutes a different phylogroup from phylogroup 1.

Phylogroup 1 consists of 34 strains. These strains are from 6 different isolation sites, all from hypersaline habitats located in Spain; this phylogroup does not contain any previously described species and could constitute a new species of the genus *Salinivibrio*. Phylogroup 2 is composed of 25 strains including the type strain of the species *S. siamensis*. These 25 strains are from different places of isolation from Spain and Puerto Rico. In the case of phylogroup 3, it consists of nine strains, including two subspecies of *S. costicola*, *S. costicola* subsp. *costicola* and *S. costicola* subsp. *alcaliphilus*. The other seven isolated strains are from different Spanish sites. As to phylogroup 4, it is composed of seven strains, one of them being the type strain of *S. proteolyticus* and another being the remaining type strain of the subspecies, *S. costicola* subsp. *vallismortis*. The other five strains are from two locations, Puerto Rico and Isla Bacuta in Spain.

Besides, we studied the phylogeny of the amino acid sequences resulting from the translation of the protein-encoding nucleotide sequences. For that purpose, the NJ tree was calculated using the Jones-Taylor-Thornton (JTT) model. As expected, the resulting tree showed a less clear separation among phylogroups/species (data not shown), because of the fact that amino acid sequences exhibit a more conservative character than nucleotide ones.

Regarding the concatenated tree, higher bootstrap values support the branches formed by the phylogroups compared to the individual gene trees, confirming the robustness of this approach. This agrees with the study of Wertz et al. (2003) that states that the concatenation of a number of gene sequences proportionally reduce the influence of aberrant signal genes and reinforce the underlying common phylogenetic signal, as showed by the increase in bootstrap values. In this study a total of four housekeeping genes were used; this number of genes was balanced between confidence of results and reduction of cost and time. Urbanczyk et al. (2007) described the new genus *Aliivibrio* to place the species *V. fischeri* and close relatives. The proposal of this new taxon was supported by a MLSA study using the concatenation of several genes (*recA*, *rpoA*, *pyrH*, *gyrB*, and 16S rRNA). Rameshkumar et al. (2008) described a novel species of the genus *Vibrio*, *V. porteresiae*, based on the phylogenetic analysis of the concatenated sequences of four genes, the 16S rRNA gene, *rpoA*, *recA*, and *pyrH*. These studies are examples of previous MLSA analysis where a small number of housekeeping genes have been used in the family *Vibrionaceae*. Concerning other halophilic bacterial taxa, a MLSA study in the family *Halomonadaceae*, which included moderately halophilic bacteria belonging to the class *Gammaproteobacteria*, demonstrated that a reduced number of housekeeping genes maintained a high resolution in the phylogenetic trees analyzed (de la Haba et al., 2012).

### *gyrB*, *recA*, *rpoA*, and *rpoD* Gene Sequence Analysis

**FIGURE 3:**
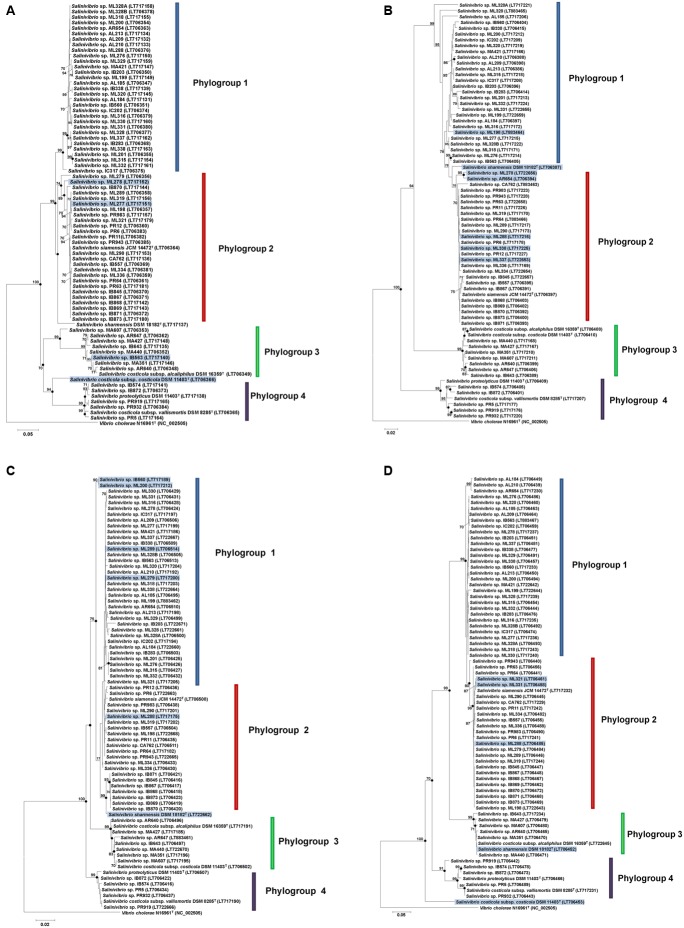
Phylogenetic reconstructions of *Salinivibrio* strains based on individual analyses of the *gyrB* **(A)**, *recA* **(B)**, *rpoA* **(C)**, and *rpoD* **(D)** genes using the neighbor-joining method. Circles indicate branches that were supported by NJ, MP, and ML algorithms. The GenBank/EMBL/DDBJ accession number of each sequence is shown in parentheses. Possible events of recombination with respect to the concatenated tree are marked in blue. Only bootstrap values above 70% are shown (1000 replications) at branches points. Bars, 0.02 **(B,C)** and 0.05 **(A,D)** expected nucleotide substitutions per site. *V. cholerae* N16961^T^ was used as an outgroup.

Separate phylogenetic trees for each housekeeping gene were constructed using NJ, ML, and MP methods; neighbor-joining trees are shown in **Figures 3A–D**. The trees exhibit in general a similar topology, even if the precise branching pattern showed some variations.

In the case of the *gyrB* tree, both phylogroups 1 and 2 as well as phylogroups 3 and 4 are sister groups, but not in the other trees, where phylogroup 3 is sister to groups 1 and 2. This could indicate a significant evolutionary difference for this gene compared to the others. Although the bootstrap support for group 3 is very high among the individual trees, their different branching patterns indicate its position related to the other groups is not clear with the current dataset and analyses. As to bootstrap branch support, in the *gyrB* tree it is 99% for phylogroups 1, 3, and 4; and 74% for phylogroup 2. In the *recA* tree it is 99% for phylogroups 3 and 4. But it is not possible to distinguish phylogroup 1 from 2, although the branch supporting both phylogroups shows a bootstrap value of 99%. In the *rpoA* tree, phylogroups 3 and 4 have a high bootstrap value of 99%, while phylogroups 1 and 2 are joined within the same branch with a value of 75%. In this case, *S. sharmensis* fell into this big phylogroup. In the case of the *rpoD* tree, phylogroups 3 and 4 have a high bootstrap value of 99%, while phylogroup 2 has a bootstrap branch support of 80% and phylogroup 1 is not well supported.

Overall, in comparison with the 16S rRNA gene tree, phylogenetic trees predicted from individual housekeeping gene sequences presented congruent phylogroups. All individual protein-encoding gene trees showed higher resolution than the 16S rRNA gene tree, although not enough to discriminate all the species identified by the concatenated gene tree. There were some poorly resolved relationships and disagreements among the trees, e.g., the relationship between phylogroups 1 and 2, where the *recA* and *rpoA* gene trees showed no clear differentiation. The relationship of phylogroup 3 is still unresolved. It could be that the close relationship between groups 1 and 2 forces together the presence of groups 3 and 4. These observations suggest that both genes (*recA* and *rpoA*) have been subjected to horizontal gene transfer (HGT) events during their evolutionary history, and a single-gene phylogeny can confound the identification of taxa. It is clear that individual protein-encoding gene phylogenies cannot be assumed automatically to indicate the appropriate evolutionary history of the organisms and should be regarded with caution.

For each individual gene the phylogeny is conserved although there are some strains that change its position in the tree. These different placements of strains in single-gene tree analyses might be consequence of the different evolution processes that undergo the genes, like recombination events, HGT or intragenomic rearrangements. These variations are marked in blue in the phylogenetic trees (**Figure 3**).

Evolutionary information was calculated for each housekeeping gene. **Table 2** shows that, in all the cases, Tajima’s *D* values were negative suggesting a recent population bottleneck followed by expansion. Absence of positive selection along with violation of the molecular clock suggested a nearly neutral mechanism for the analyzed housekeeping gene evolution (Liao et al., 2017).

**Table 2:** Descriptive analysis of nucleotide sequence data for each housekeeping gene.

Gene	No. of Polymorphic sites	No. of Mutations	ð^a^	k^b^	Tajima’s *D* value
*gyrB*	186	254	0.12241	39.29309	-0.81207
*recA*	196	262	0.07201	41.55171	-0.77874
*rpoA*	103	116	0.02985	13.99789	-1.13163
*rpoD*	304	387	0.07577	46.44947	-0.87036

*^a^Average pairwise nucleotide diversity per site. ^b^Average pairwise nucleotide differences per sequence.*

### DNA–DNA Hybridization

DNA–DNA hybridization data have been used since the 1960s to determine the relatedness between strains and is still considered as the most important criterion in the delineation of prokaryotic species, as it was one of the few universally applicable techniques available that could offer truly genome-wide comparisons between organisms (Al-Saari et al., 2015; Glaeser and Kämpfer, 2015; Dubert et al., 2016a,b). In this study, 25 selected strains were used for DDH experiments, including the type strains of *S. costicola* subsp. *alcaliphilus* DSM 16359^T^, *S. costicola* subsp. *costicola* DSM 11403^T^, *S. costicola* subsp. *vallismortis* DSM 8285^T^, *S. proteolyticus* DSM 11403^T^, *S. sharmensis* DSM 18182^T^ and *S. siamensis* JCM 14472^T^, along with additional representative strains of each phylogroup as determined by the concatenated gene phylogeny. **Table 3** shows the DDH values for *Salinivibrio* strains included in this study. A representative strain of each phylogroup was selected for radioactive labeling to carry out the DDH analyses (AL184^T^ for phylogroup 1, *S. siamensis* JCM 14472^T^ for phylogroup 2, *S. costicola.* subsp. *costicola* DSM 11403^T^ for phylogroup 3, and *S. costicola* subsp. *vallismortis* DSM 8285^T^ for phylogroup 4). The DDH percentage values for strains within the same phylogroup were always above 70%, a value established as cut-off for species delineation (Wayne et al., 1987; Stackebrandt and Goebel, 1994), confirming that they belong to the same species. DDH analyses among phylogroups always showed values lower than 70%, indicating that each phylogroup constitutes a different species. In addition, DDH *in silico* was calculated using the information from the draft genomes available from the GenBank database (**Table 3**). Comparison between experimental and *in silico* DDH values shows that, independently of the percentages obtained from both approaches, there is an agreement on their biological significance, with percentages of hybridization higher than 70% when strains of the same phylogroup were analyzed, and lower than this value for strains belonging to different phylogroups.

**Table 3:** DNA–DNA hybridization within *Salinivibrio* phylogroups and among representative strains of each phylogroup.

DDH values within *Salinivibrio* phylogroups			Percentage of DDH with respect to^∗^	
Phylogroup 1			*Salinivibrio* sp. AL184	
*Salinivibrio* sp. IB560			72% (86.3% ± 1.94)	
*Salinivibrio* sp. IC202			98% (83.6% ± 1.61)	
*Salinivibrio* sp. MA421			95% (84.5% ± 1.91)	
*Salinivibrio* sp. ML331			72% (86.7% ± 1.67)	

Phylogroup 2			*S. siamensis* JCM 14472^T^	

*Salinivibrio* sp. IB868			98% (89.1% ± 8.19)	
*Salinivibrio* sp. IB870			97% (89.1% ± 8.19)	
*Salinivibrio* sp. ML198			80% (90.0% ± 6.41)	
*Salinivibrio* sp. ML290			95% (85.4% ± 5.55)	
*Salinivibrio* sp. PR6			86% (89.5% ± 6.47)	

Phylogroup 3			*S. costicola* subsp. *costicola* DSM 11403^T^	

*Salinivibrio* sp. AR640			73% (83.8% ± 1.77)	
*Salinivibrio* sp. AR647			93% (83.6% ± 2.56)	
*Salinivibrio* sp. IB643			72% (80.4% ± 1.77)	
*Salinivibrio* sp. MA351			74% (85.6% ± 1.60)	
*Salinivibrio* sp. MA427			100% (82.4% ± 2.95)	
*Salinivibrio* sp. MA440			78% (84.2% ± 2.47)	
*Salinivibrio* sp. MA607			81% (86.5% ± 1.73)	
*S. costicola* subsp. *alcaliphilus* DSM 16359^T^			72% (88.2% ± 2.15)	

Phylogroup 4			*S. costicola* subsp. *vallismortis* DSM 8285^T^	

*Salinivibrio* sp. IB872			98% (76.7% ± 6.64)	
*Salinivibrio* sp. PR5			76% (91.5% ± 4.00)	
*Salinivibrio* sp. PR919			81% (94.5% ± 4.00)	
*S. proteolyticus* DSM 11403^T^			81% (81.1% ± 6.34)	

**DDH values among representative strains of each phylogroup**	**1**	**2**	**3**	**4**

*Salinivibrio* sp. AL184 (Phylogroup 1)	100	35	17	44
*S. siamensis* JCM 14472^T^ (Phylogroup 2)	17	100	47	23
*S. sharmensis* DSM 18182^T^	8	60	30	29
*S. costicola* subsp. *costicola* DSM 11403^T^(Phylogroup 3)	28	17	100	15
*S. costicola* subsp. *vallismortis* DSM 8285^T^ (Phylogroup 4)	37	34	17	100

*Representative strains of each phylogroup: 1, Salinivibrio sp. AL184^T^; 2, S. siamensis JCM 14472^T^; 3, S. costicola subsp. costicola DSM 11403^T^; 4, S. costicola subsp. vallismortis DSM 8285^T^. ^∗^Values obtained by the conventional DDH method as well as the in silico DDH (shown in parenthesis).*

### Correlation and Validation of DDH Data with MLSA Study

**FIGURE 4:**
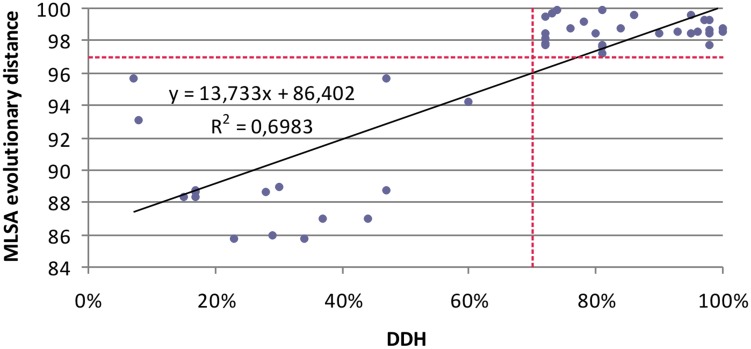
Relationship between five-gene MLSA evolutionary distance and DDH data for *Salinivibrio*. Each solid diamond represents *Salinivibrio* strains plotted against the DDH value of the strains (*x*-axis) versus the MLSA evolutionary distance (*y*-axis).

Few studies have compared MLSA sequence data with DDH data. To evaluate the resolution of the MLSA scheme and calibrate MLSA sequence similarity to the gold-standard for circumscribing taxonomic species, we plotted each measured pairwise value and determined the linear Pearson’s product–moment correlation coefficient for the individual 16S rRNA and housekeeping genes, and the four gene concatenation (which gave the highest correlation; 0.84). This result supports the conclusion of Konstantinidis et al. (2006) that a concatenation of genes, rather than a single gene, more accurately predicts inter-organismal relationships. The coefficients obtained were as low as 0.48 for *gyrB*, followed by 0.55 for *rpoD*, 0.55 for 16s rRNA gene, 0.78 for *rpoA*, 0.8 for *recA*. The relationship between DDH and MLSA concatenated gene distance was described by a linear regression model. **Figure 4** shows that the DDH value correlated (*r*^2^= 0.69) with the concatenated gene evolutionary distance, and the 70% DDH value for the current species definition corresponded to an MLSA distance of 97%. Consequently, the four-gene MLSA similarity of 97% could be considered as the cut-off for species within the genus *Salinivibrio*, suggesting that species with four-gene sequence similarity equal or below 97% should be assigned to different species. With this calibration, the use of MLSA in describing new *Salinivibrio* species provides a robust species delineation and avoids the necessity for performing DDH in future taxonomic studies on the genus.

Our study indicates that the concatenation of housekeeping genes provide a robust species delineation that is at least equivalent to DDH. Although the housekeeping genes may also be affected by HGT, in our study their concatenated sequences, in contrast to the 16S rRNA gene sequence analysis, clearly allow the differentiation among phylogroups and, therefore, the adequate delineation at the *Salinivibrio* species-level. MLSA, like DDH, is a suitable technique for species circumscription and, additionally, for assessing relationships at the *Salinivibrio* intraspecies level (Pascual et al., 2010; Marti and Balcázar, 2015).

**FIGURE 5:**
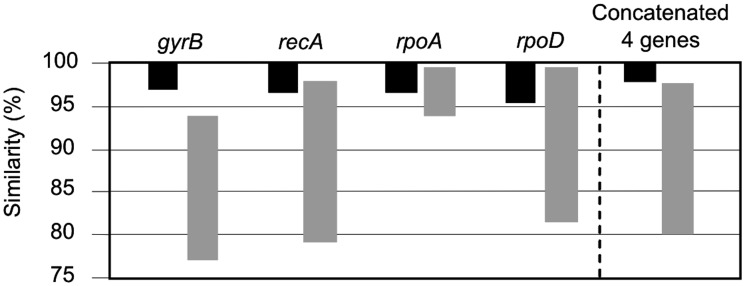
Taxonomic resolution based on the intra-phylogroup (black bars) and inter-phylogroup (gray bars) ranges of similarity (%).

The intra-phylogroup gene sequence similarities were 97.0–100%, and 96.2–100%, 96.5–100% and 96.0–100% for the *gyrB*, *recA*, *rpoA*, and *rpoD*, respectively. The inter-phylogroup gene sequence similarities were 77.7–93.3%, 79.7–98.2%, 94.1–99.8%, and 81.4–99.7% for *gyrB*, *recA*, *rpoA*, and *rpoD*, respectively (**Figure 5**). In the case of the concatenated tree, the range of intraphylogroup sequence similarity was 97.9–100% and the range of interphylogroup sequence similarity was 80.0–97.5% (**Figure 5**). The concatenation of the four proposed housekeeping genes is the best way to proceed in future studies due to the lack of resolution of single gene analyses and their distinct possibilities of HGT.

In summary, a *Salinivibrio* classification scheme is proposed that uses: (1) partial sequencing of the 16S rRNA gene for genus-level identification and (2) sequencing and concatenation of the *gyrB, recA, rpoA*, *rpoD* housekeeping genes for species-level discrimination with a cut-off value of 97% for MLSA study.

### Genomic Fingerprinting

Extremophiles often demonstrate patterns of evolution that mirror their geographic origins, and MLSA has been reported to be capable of discerning biogeographic influences, even among strains belonging to the same species (Papke et al., 2003; Whitaker et al., 2003; Rosselló-Mora et al., 2008). However, our multilocus analysis did not convincingly recover a geographic pattern. Perhaps this was because most of the strains came from regions around Spain providing easy migration between sites. Alternatively, MLSA was not sensitive enough to separate according to location the *Salinivibrio* strains. Previous studies have shown that genomic fingerprinting is powerful in discovering geographic patterns among strains (Cho and Tiedje, 2000), so we applied a similar analysis to our *Salinivibrio* strain collection. Additionally, genomic fingerprinting is a useful typing method which permits the assessment of the non-clonality of the isolated strains.

**FIGURE 6:**
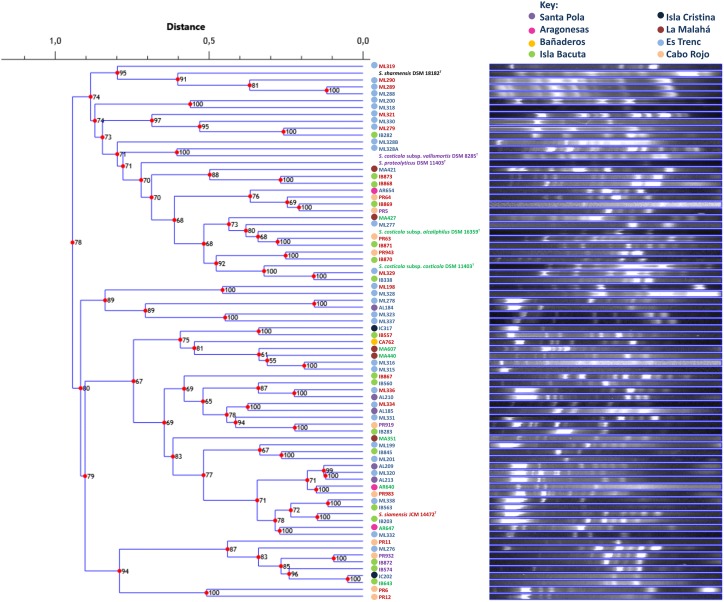
UPGMA dendrogram comparing banding patterns between isolated and type strains of the genus *Salinivibrio*. The numbers displayed at the nodes represent the cophenetic correlation coefficients. Each place of isolation is represented with a circle of different color. Strain names are colored according to the phylogroup they belong to.

The repeatability of banding patterns was tested on the 76 strains used in this study. This technique was performed in triplicate and it was observed that there was reproducibility in the banding patterns obtained for each tested strain. Banding patterns for the 76 strains were assessed using software Phoretix 1D Pro that made a UPGMA dendrogram of the genomic fingerprints (**Figure 6**). Genomic fingerprint analysis was run individually for each environmental isolate as well as for each reference type strain. Our results demonstrated that closely related strains within a single phylogroup displayed numerous banding pattern variations, in some cases dissimilar to each other. Unfortunately, the rep-PCR fingerprinting did not reliably differentiate the same phylogroups obtained by MLSA, and further, strains did not form groups according to their place of isolation. Because the genomic fingerprinting technique did not recover phylogroups, it was difficult to additionally conclude that, in the case of the genus *Salinivibrio*, endemism does not exist. The fingerprinting analysis did point to highly dynamic processes that generate great genomic variation between *Salinivibrio* strains, suggesting gene gain and loss and/or genomic rearrangements may have played significant roles in their recent evolution and may have obscured evidence for geographic patterning, if it existed. Moreover, the fingerprinting analyses confirmed that the environmental isolates are in fact different strains.

## Conclusion

We have demonstrated that MLSA is a good alternative for DDH and 16S rRNA gene sequencing in taxonomic studies of the genus *Salinivibrio* which should alleviate potential pitfalls associated with those latter techniques, and provide a robust classification scheme. Nevertheless, as previously discussed, a critical issue is the election of the protein-encoding genes, since not all genes tested in this study so far produced a successful result. This study also emphasizes how important is to include a reasonable number of strains that represent real populations for each species, and not only type strains.

In addition to phylogenetic analysis, researchers intend to propose MLSA as a replacement for DDH assays, for which it is very important to validate the MLSA scheme versus DDH. Results in this study show, indeed, that there is a correlation between MLSA and DDH assays, allowing us to establish a cut-off value of 97% for MLSA, so that strains sharing MLSA similarity values below 97% can be regarded as different *Salinivibrio* species. Regarding to the 16S rRNA gene, this analysis indicates that inclusion of 16S rRNA gene sequences is not necessary for reconstructing the *Salinivibrio* phylogeny on the basis of MLSA because the 16S rRNA tree does not allow to distinguish between phylogroups. In the case of the genus *Salinivibrio*, good housekeeping genes with potential enough to classify and identify strains are the *gyrB, recA, rpoA*, and *rpoD*. For taxonomic identification purposes of new isolates, a general strategy could be made up of: (i) initial partial sequencing of the 16S rRNA gene for genus-level identification, but, in general, not able to distinguish between closely related species; (ii) sequencing and concatenation of four housekeeping genes (*gyrB, recA, rpoA*, and *rpoD*) for species-level discrimination; (iii) when the concatenated MLSA similarity values among a new isolate and other *Salinivibrio* strains are above the 97% cut-off, then DDH experiments are required in order to assess the placement of the new isolate as a new species of *Salinivibrio*, or as new strain of an already described species. According to this study, the genus *Salinivibrio* would need a reclassification since some incongruities have been observed, such that in the phylogroup 3 a species and a subspecies fall in the same phylogroup, as well as the description of a possible new species for strains of phylogroup 1; this phylogroup was not resolved with the 16S rRNA gene. In addition, as it has been observed in phylogroup 3, neither DDH nor the MLSA study are able to differentiate at the subspecies level.

## Author Contributions

Conceived and designed the study: AV, CS-P, RRH, and RTP; designed and performed the acquisition of environmental isolates: CL-H, RRH, and AV; performed the microbial analyses: CL-H, RRH, and RTP; analyzed and interpreted the data: CL-H, RRH, CS-P, RTP, and AV; discussed the phylogenetics considerations: CL-H, RRH, CS-P, RTP, and AV; drafted the paper: CL-H and RRH; critically revised the manuscript: CL-H, RRH, CS-P, RTP, and AV. All authors read and approved the final manuscript.

## Conflict of Interest Statement

The authors declare that the research was conducted in the absence of any commercial or financial relationships that could be construed as a potential conflict of interest.
