# Supplementary material for: Assessment of MultiLocus Sequence Analysis As a Valuable Tool for the Classification of the Genus Salinivibrio
Source: Front Microbiol. 2017 Jun 22;8:1107. doi: 10.3389/fmicb.2017.01107 (PMC5479898; doi:10.3389/fmicb.2017.01107)
Supplement: Supplementary file 1 [file Table_1.doc]

Supplementary Material

**Clara López-Hermoso, Rafael R. de la Haba, Cristina Sánchez-Porro,** **R. Thane Papke and Antonio Ventosa^*^**

*** Correspondence:** ventosa@us.es

# Supplementary Data

**MATERIAL AND METHODS**

**Primers design**

Primers for *gyrB* were designed specifically for this work using the corresponding sequences derived from the whole-genome sequences of related members of the class *Gammaproteobacteria*: *Aliivibrio salmonicida* (Hjerde et al., 2008), *Escherichia coli* (Blattner et al., 1997; Riley et al., 2006), *Listonella anguillarum* (Naka et al., 2011), *Photobacterium profundum* (Vezzi et al., 2005), *Vibrio alginolyticus* (Cao et al., 2013), *Vibrio anguillarum* (Li et al., 2013), *Vibrio cholerae* (Pérez Chaparro et al., 2011), *Vibrio harveyi* (Huang et al., 2012), *Vibrio parahaemolyticus* (Liu et al., 2013), *Vibrio splendidus* (J. Craig Venter Institute, accession no.: NZ_AAMR00000000) and *Vibrio vulnificus* (Wang et al., 2012). The *gyrB* gene sequences were aligned using CLUSTAL_X 2.1 (Larkin et al., 2007) and compared using the BOXSHADE 3.21 program (<http://www.ch.embnet.org/software/BOX_form.html>) in order to identify conserved regions for the development of suitable primers.

1. **Supplementary Figures and Tables**

**Supplementary Table 1.** Salinity and pH of the sampling sites studied.

| **Place of isolation** | **Salinity (%)** | **pH** |
| --- | --- | --- |
| Santa Pola, Alicante (Spain) | 5, 12, 19 | 7.4, 8.0 |
| Aragonesas, Huelva (Spain) | 6, 16 | 7.2, 8.3 |
| Bañaderos, Gran Canaria (Spain) | 6.5, 8, 16 | 7.2, 7.4 |
| Isla Bacuta, Huelva (Spain) | 5, 7, 8.5, 11, 17, 20 | 7.1, 8.1 |
| Isla Cristina, Huelva (Spain) | 17, 19, 21 | 7.5, 7.6 |
| La Malahá, Granada (Spain) | 5, 10, 13.5, 15 | 7.2, 7.6 |
| Es Trenc, Mallorca (Spain) | 12 | 7.4 |
| Cabo Rojo (Puerto Rico) | 8, 16, 19 | 7.1, 8.0 |

**Supplementary Table 2**. Strains used in this study, their date, place and source of isolation and GenBank/EMBL/DDBJ accession numbers for the 16S rRNA, *gyrB*, *recA*, *rpoA* and *rpoD* genes.

| **Strain^a^** | **Date of isolation/publishing** | **Place of isolation** | **Isolation source** | **16S rRNA** | ***gyrB*** | ***recA*** | ***rpoA*** | ***rpoD*** |
| --- | --- | --- | --- | --- | --- | --- | --- | --- |
| *S. costicola* subsp. *costicola* CECT 4059^T^ | July 1996 | Australia | Cured meat | LT722673 (X74699)^b^ | LT706366 | LT706410 | LT706502 | LT706453 |
| *S. costicola* subsp. *alcaliphilus* DSM 19052^T^ | September 2004 | Campania (Italy) | Saline lake | LT627802 (AJ640132) ^b^ | LT706349 | LT706408 | LT717191 | LT722645 |
| *S. costicola* subsp. *vallismortis* DSM 8285^T^ | March 2000 | Death Valley (USA) | Hypersaline pond | LT722672 (AF057016)^b^ | LT706365 | LT717207 | LT717190 | LT717231 |
| *S. proteolyticus* DSM 19052^T^ | May 2008 | Bakhtegan Lake (Iran) | Saline lake | LT627803 (DQ092443)^b^ | LT717138 | LT706409 | LT706507 | LT706466 |
| *S. sharmensis* DSM 18182^T^ | May 2010 | Ras Mohammed Park (Egypt) | Saline lake | LT627804 (AM279734)^b^ | LT717137 | LT706387 | LT722662 | LT706452 |
| *S. siamensis* JCM 14472^T^ | April 2009 | Nakornnayok (Thailand) | Fermented fish product | LT627805 (AB285018)^b^ | LT706364 | LT706411 | LT706508 | LT717232 |
| **AL184** | November 2011 | Santa Pola, Alicante (Spain) | Solar saltern | LT627515 | LT717131 | LT706397 | LT722660 | LT706449 |
| **AL185** | November 2011 | Santa Pola, Alicante (Spain) | Solar saltern | LT627737 | LT706347 | LT717206 | LT706495 | LT706463 |
| **AL209** | November 2011 | Santa Pola, Alicante (Spain) | Solar saltern | LT627738 | LT717132 | LT706398 | LT706506 | LT706464 |
| **AL210** | November 2011 | Santa Pola, Alicante (Spain) | Solar saltern | LT722647 | LT717133 | LT706388 | LT717192 | LT706439 |
| **AL213** | November 2011 | Santa Pola, Alicante (Spain) | Solar saltern | LT627740 | LT717134 | LT706386 | LT717198 | LT706450 |
| **AR640** | May 2013 | Aragonesas, Huelva (Spain) | Solar saltern | LT627741 | LT706348 | LT706399 | LT706496 | LT706465 |
| **AR647** | May 2013 | Aragonesas, Huelva (Spain) | Solar saltern | LT627742 | LT706362 | LT706406 | LT883461 | ND^c^ |
| **AR654** | May 2013 | Aragonesas, Huelva (Spain) | Solar saltern | LT627743 | LT706363 | LT706394 | LT706510 | LT717230 |
| **CA762** | September 2013 | Bañaderos, Gran Canaria (Spain) | Solar saltern | LT627744 | LT717136 | LT883463 | LT706511 | LT717229 |
| **IB203** | October 2011 | Isla Bacuta, Huelva (Spain) | Solar saltern | LT627745 | LT706350 | LT706396 | LT722671 | LT706451 |
| **IB283** | October 2011 | Isla Bacuta, Huelva (Spain) | Solar saltern | LT627739 | LT706368 | LT706414 | LT706503 | LT706476 |
| **IB338** | October 2011 | Isla Bacuta, Huelva (Spain) | Solar saltern | LT722650 | LT717139 | LT706415 | LT706509 | LT706477 |
| **IB557** | January 2013 | Isla Bacuta, Huelva (Spain) | Solar saltern | LT627747 | LT706369 | LT706395 | LT706504 | LT706455 |
| **IB560** | January 2013 | Isla Bacuta, Huelva (Spain) | Solar saltern | LT627748 | LT706351 | LT706404 | LT717189 | LT717233 |
| **IB563** | May 2013 | Isla Bacuta, Huelva (Spain) | Solar saltern | LT722646 | LT717140 | LT706405 | LT706513 | LT883467 |
| **IB574** | May 2013 | Isla Bacuta, Huelva (Spain) | Solar saltern | LT627749 | LT717141 | LT706412 | LT717188 | LT706478 |
| **IB643** | May 2013 | Isla Bacuta, Huelva (Spain) | Solar saltern | LT627750 | LT717135 | LT706389 | LT706497 | LT717234 |
| **IB845** | September 2013 | Isla Bacuta, Huelva (Spain) | Solar saltern | LT722648 | LT706370 | LT722657 | LT706416 | LT706447 |
| **IB867** | September 2013 | Isla Bacuta, Huelva (Spain) | Solar saltern | LT627751 | LT706371 | LT706391 | LT706417 | LT706448 |
| **IB868** | September 2013 | Isla Bacuta, Huelva (Spain) | Solar saltern | LT627752 | LT717142 | LT706403 | LT706418 | LT706467 |
| **IB869** | September 2013 | Isla Bacuta, Huelva (Spain) | Solar saltern | LT627753 | LT717143 | LT706402 | LT706419 | LT706462 |
| **IB870** | September 2013 | Isla Bacuta, Huelva (Spain) | Solar saltern | LT627754 | LT717144 | LT706392 | LT706420 | LT706472 |
| **IB871** | September 2013 | Isla Bacuta, Huelva (Spain) | Solar saltern | LT627755 | LT706372 | LT706393 | LT706421 | LT706468 |
| **IB872** | September 2013 | Isla Bacuta, Huelva (Spain) | Solar saltern | LT627756 | LT706373 | LT706401 | LT706422 | LT706473 |
| **IB873** | September 2013 | Isla Bacuta, Huelva (Spain) | Solar saltern | LT627757 | LT717180 | LT706400 | LT706423 | LT706469 |
| **IC202** | October 2011 | Isla Cristina, Huelva (Spain) | Solar saltern | LT627758 | LT706374 | LT717209 | LT717194 | LT706459 |
| **IC317** | October 2011 | Isla Cristina, Huelva (Spain) | Solar saltern | LT627759 | LT706375 | LT717208 | LT717197 | LT706474 |
| **MA351** | November 2012 | La Malahá, Granada (Spain) | Solar saltern | LT627760 | LT717146 | LT717210 | LT717196 | LT706470 |
| **MA421** | November 2012 | La Malahá, Granada (Spain) | Solar saltern | LT627761 | LT717147 | LT717166 | LT717186 | LT722642 |
| **MA427** | November 2012 | La Malahá, Granada (Spain) | Solar saltern | LT627762 | LT717148 | LT717167 | LT717185 | LT706479 |
| **MA440** | November 2012 | La Malahá, Granada (Spain) | Solar saltern | LT627763 | LT706352 | LT717168 | LT722670 | LT706471 |
| **MA607** | May 2013 | La Malahá, Granada (Spain) | Solar saltern | LT627764 | LT706353 | LT717211 | LT717195 | LT706480 |
| **ML198** | November 2011 | Es Trenc, Mallorca (Spain) | Solar saltern | LT627765 | LT706357 | LT883464 | LT722668 | LT722643 |
| **ML199** | November 2011 | Es Trenc, Mallorca (Spain) | Solar saltern | LT627766 | LT717149 | LT722659 | LT883462 | LT722644 |
| **ML200** | November 2011 | Es Trenc, Mallorca (Spain) | Solar saltern | LT627767 | LT706354 | LT717212 | LT717184 | LT706494 |
| **ML201** | November 2011 | Es Trenc, Mallorca (Spain) | Solar saltern | LT627768 | LT706355 | LT717213 | LT706425 | ND^c^ |
| **ML276** | November 2011 | Es Trenc, Mallorca (Spain) | Solar saltern | LT627769 | LT717150 | LT717214 | LT706426 | LT706486 |
| **ML277** | November 2011 | Es Trenc, Mallorca (Spain) | Solar saltern | LT627770 | LT717151 | LT717215 | LT717199 | LT717236 |
| **ML278** | November 2011 | Es Trenc, Mallorca (Spain) | Solar saltern | LT627771 | LT717152 | LT722656 | LT706424 | LT717237 |
| **ML279** | November 2011 | Es Trenc, Mallorca (Spain) | Solar saltern | LT627772 | LT706356 | ND^c^ | LT717200 | LT706484 |
| **ML288** | November 2011 | Es Trenc, Mallorca (Spain) | Solar saltern | LT627773 | LT706376 | LT717216 | LT717175 | LT706485 |
| **ML289** | November 2011 | Es Trenc, Mallorca (Spain) | Solar saltern | LT627774 | LT706358 | LT717217 | LT706514 | LT706446 |
| **ML290** | November 2011 | Es Trenc, Mallorca (Spain) | Solar saltern | LT627775 | LT717153 | LT717173 | LT717201 | LT706445 |
| **ML315** | November 2011 | Es Trenc, Mallorca (Spain) | Solar saltern | LT722649 | LT717154 | LT717218 | LT706427 | LT706454 |
| **ML316** | November 2011 | Es Trenc, Mallorca (Spain) | Solar saltern | LT627776 | LT706379 | LT717172 | LT706428 | LT717235 |
| **ML318** | November 2011 | Es Trenc, Mallorca (Spain) | Solar saltern | LT627777 | LT717155 | LT717171 | LT717203 | LT717243 |
| **ML319** | November 2011 | Es Trenc, Mallorca (Spain) | Solar saltern | LT627778 | LT717156 | LT717170 | LT717202 | LT717244 |
| **ML320** | November 2011 | Es Trenc, Mallorca (Spain) | Solar saltern | LT627779 | LT717145 | LT717219 | LT717204 | LT706460 |
| **ML321** | November 2011 | Es Trenc, Mallorca (Spain) | Solar saltern | LT627780 | LT717179 | ND^c^ | LT717205 | LT706461 |
| **ML328** | November 2011 | Es Trenc, Mallorca (Spain) | Solar saltern | LT627782 | LT706377 | LT883465 | LT722661 | LT717239 |
| **ML328A** | November 2011 | Es Trenc, Mallorca (Spain) | Solar saltern | LT627783 | LT717158 | LT717221 | LT706500 | LT706493 |
| **ML328B** | November 2011 | Es Trenc, Mallorca (Spain) | Solar saltern | LT627784 | LT706378 | LT717222 | LT706505 | LT706492 |
| **ML329** | November 2011 | Es Trenc, Mallorca (Spain) | Solar saltern | LT627785 | LT717159 | ND^c^ | LT706499 | LT706491 |
| **ML330** | November 2011 | Es Trenc, Mallorca (Spain) | Solar saltern | LT627786 | LT717160 | ND^c^ | LT706429 | LT717240 |
| **ML331** | November 2011 | Es Trenc, Mallorca (Spain) | Solar saltern | LT627787 | LT706380 | LT722655 | LT706431 | LT706458 |
| **ML332** | November 2011 | Es Trenc, Mallorca (Spain) | Solar saltern | LT627788 | LT717161 | LT717224 | LT706432 | LT706444 |
| **ML334** | November 2011 | Es Trenc, Mallorca (Spain) | Solar saltern | LT722651 | LT706381 | LT722654 | LT706433 | LT706482 |
| **ML336** | November 2011 | Es Trenc, Mallorca (Spain) | Solar saltern | LT627789 | LT706359 | LT717169 | LT706430 | LT706488 |
| **ML337** | November 2011 | Es Trenc, Mallorca (Spain) | Solar saltern | LT627790 | LT717162 | LT722653 | LT722667 | LT706481 |
| **ML338** | November 2011 | Es Trenc, Mallorca (Spain) | Solar saltern | LT722652 | LT717163 | LT717225 | LT722664 | LT706457 |
| **PR11** | September 2013 | Cabo Rojo (Puerto Rico) | Solar saltern | LT627793 | LT706382 | LT717226 | LT706435 | LT717242 |
| **PR12** | September 2013 | Cabo Rojo (Puerto Rico) | Solar saltern | LT627794 | LT706360 | LT717227 | LT706436 | ND^c^ |
| **PR5** | September 2013 | Cabo Rojo (Puerto Rico) | Solar saltern | LT627791 | LT717164 | LT717177 | LT706434 | LT706489 |
| **PR6** | September 2013 | Cabo Rojo (Puerto Rico) | Solar saltern | LT627792 | LT706383 | LT717178 | LT722663 | LT717241 |
| **PR63** | September 2013 | Cabo Rojo (Puerto Rico) | Solar saltern | LT627796 | LT717181 | LT722658 | ND^c^ | LT706456 |
| **PR64** | September 2013 | Cabo Rojo (Puerto Rico) | Solar saltern | LT627797 | LT706361 | LT883466 | LT717182 | LT706441 |
| **PR919** | September 2013 | Cabo Rojo (Puerto Rico) | Solar saltern | LT627798 | LT717165 | LT717176 | LT722666 | LT706442 |
| **PR932** | September 2013 | Cabo Rojo (Puerto Rico) | Solar saltern | LT627799 | LT706384 | LT717220 | LT706437 | LT706443 |
| **PR943** | September 2013 | Cabo Rojo (Puerto Rico) | Solar saltern | LT627800 | LT706385 | LT717228 | LT722665 | LT706440 |
| **PR983** | September 2013 | Cabo Rojo (Puerto Rico) | Solar saltern | LT627801 | LT717157 | LT717223 | LT706438 | LT706490 |

^a^Strains isolated in this study are marked in bold; ^b^accession numbers of type strains from previous studies; ^c^ND, not determined.

**REFERENCES**

Blattner, F. R., Plunkett, G., Bloch, C. A., Perna, N. T., Burland, V., Riley, M., et al. (1997). The complete genome sequence of *Escherichia coli* K-12. *Science* 277, 1453-1462. doi: 10.1126/science.277.5331.1453

Cao, Y., Liu, X. F., Zhang, H. L., Chen, Y. J., and Hu, C. J. (2013). Draft genome sequence of the human-pathogenic bacterium *Vibrio alginolyticus* E0666. *Genome Announc.* 1, e00686-13. doi: 10.1128/genomeA.00686-13

Hjerde, E., Lorentzen, M. S., Holden, M. T., Seeger K., Paulsen, S., Bason, N., et al. (2008). The genome sequence of the fish pathogen *Aliivibrio salmonicida* strain LFI1238 shows extensive evidence of gene decay. *BMC Genomics* 9, 616. doi: 10.1186/1471-2164-9-616

Huang, Y., Jian, J., Lu, Y., Cai, S., Wang, B., Tang, J., et al. (2012). Draft genome sequence of the fish pathogen *Vibrio harveyi* strain ZJ0603*. J. Bacteriol.* 194, 6644-66455. doi: 10.1128/JB.01759-12

Larkin, M.A., Blackshields, G., Brown, N. P., Chenna, R., McGettigan, P. A., McWilliam, H., et al. (2007). Clustal W and Clustal X version 2.0. *Bioinformatics* 23, 2947-2948. doi: https://doi.org/10.1093/bioinformatics/btm404

Li, G., Mo, Z., Li, J., Xiao, P., and Hao, B. (2013). Complete genome sequence of *Vibrio anguillarum* M3, a serotype O1 strain isolated from japanese flounder in China. *Genome Announc.* 1, e00769-13. doi: 10.1128/genomeA.00769-13

Liu, M., and Chen, S. (2013). Draft genome sequence of *Vibrio parahaemolyticus* V110, isolated from shrimp in Hong Kong. *Genome Announc.* 1, e00300-13. doi: 10.1128/genomeA.00300-13

Naka, H., Dias, G.M., Thompson, C. C., Dubay, C., Thompson, F. L., and Crosa, J. H. (2011). Complete genome sequence of the marine fish pathogen *Vibrio anguillarum* harboring the pJM1 virulence plasmid and genomic comparison with other virulent strains of *V. anguillarum* and *V. ordalii*. *Infect. Immun.* 79, 2889-2900. doi: 10.1128/IAI.05138-11

Pérez Chaparro, P. J., McCulloch, J. A., Cerdeira, L. T., Al-Dilaimi, A., Canto de Sá, L. L., de Oliveira, R., et al. (2011). Whole genome sequencing of environmental *Vibrio cholerae* O1 from 10 nanograms of DNA using short reads*. J. Microbiol. Methods* 87, 208-212. doi: 10.1016/j.mimet.2011.08.003

Riley, M., Abe, T., Arnaud, M. B., Berlyn, M. K., Blattner, F. R, Chaudhuri, R. et al. (2006). *Escherichia coli* K-12: a cooperatively developed annotation snapshot--2005. *Nucleic Acids Res.* 34, 1-9. doi: 10.1093/nar/gkj405

Vezzi, A., Campanaro, S., D'Angelo, M., Simonato, F., Vitulo, N., Lauro, F. M., et al. (2005). Life at depth: *Photobacterium profundum* genome sequence and expression analysis. *Science* 307, 1459-1461. doi: 10.1126/science.1103341

Wang, Z. G., Wu, Z., Xu, S. L., and Zha, J. (2012). Genome sequence of the human-pathogenetic bacterium *Vibrio vulnificus* B2. *J. Bacteriol.* 194, 7019. doi: 10.1128/JB.01955-12
